# Protective Effects of NaHS/miR-133a-3p on Lipopolysaccharide-Induced Cardiomyocytes Injury

**DOI:** 10.1155/2023/2566754

**Published:** 2023-12-08

**Authors:** Yi-Mei Jin, Ai-Rong Huang, Mei-qian Yu, Wan-Ding Ye, Xiao-guang Hu, Hua-min Wang, Zhi-wei Xu, Dong-shi Liang

**Affiliations:** Department of Pediatrics, Second Affiliated Hospital & Yuying Children's Hospital of Wenzhou Medical University, Wenzhou 325027, China

## Abstract

**Objective:**

The aim of this study was to investigate the effects of sodium hydrosulfide (NaHS) on Lipopolysaccharide (LPS)-induced cardiomyocyte injury in H9c2 cells.

**Methods:**

H9c2 cardiomyocytes cultivated with medium containing 10 *μ*g/mL LPS were used to recapitulate the phenotypes of those in sepsis. Two sequential experiments were performed. The first contained a control group, a LPS group, and a LPS + NaHS group, with the aim to assure the protective effects of NaHS on LPS-treated cardiomyocytes. The second experiment added a fourth group, the LPS + NaHS + miR-133a-3p inhibition group, with the aim to preliminarily explore whether miR-133-3p exerts a protective function downstream of NaHS. The adenosine triphosphate (ATP) kit was used to detect ATP content; real-time quantitative polynucleotide chain reaction (qPCR) was used to measure the levels of mammalian targets of rapamycin (mTOR), AMP-dependent protein kinase (AMPK), and miR-133a-3p, and Western blot (WB) was used to detect protein levels of mTOR, AMPK, myosin-like Bcl2 interacting protein (Beclin-1), microtubule-associated protein 1 light chain 3 (LC3I/II), and P62 (sequestosome-1, sqstm-1/P62).

**Results:**

Compared with the control group, the expressions of miR-133a-3p (*P* < 0.001), P62 (*P* < 0.001), and the content of ATP (*P* < 0.001) decreased, while the expressions of Beclin-1 (*P* = 0.023) and LC3I/II (*P* = 0.048) increased in the LPS group. Compared with the LPS group, the expressions of miR-133a-3p (*P* < 0.001), P62 (*P* < 0.001), and the content of ATP (*P* < 0.001) in the NaHS + LPS group increased, while the expressions of Beclin-1 (*P* = 0.023) and LC3I/II (*P* = 0.022) decreased. Compared with the NaHS + LPS group, the expression levels of miR-133a-3p (*P* < 0.001), P62 (*P* = 0.001), and the content of ATP (*P* < 0.001) in the LPS + NaHS + miR-133a-3p inhibition group were downregulated, and the expression levels of Beclin-1 (*P* = 0.012) and LC3I/II (*P* = 0.010) were upregulated. The difference was statistically significant. There was no significant difference in the expression of AMPK and mTOR between groups.

**Conclusion:**

Our research demonstrated that NaHS relieved LPS-induced myocardial injury in H9c2 by promoting the expression of miR-133a-3p, inhibiting autophagy in cardiomyocytes, and restoring cellular ATP levels.

## 1. Introduction

Sepsis-induced myocardial dysfunction causes the mortality rate to increase [1–3].

However, how sepsis influences myocardial and underlying mechanisms remains largely unknown. Hydrogen sulfide is an important endogenous biological signaling molecule in mammals and plays an important role in the physiological and pathophysiological processes of the cardiovascular system [4]. Sodium hydrosulfide (NaHS) is an important exogenous donor of hydrogen sulfide [5]. NaHS is involved in cellular autophagy through multiple pathways, which inhibits autophagy by activation of phosphatidylinositol-3-kinase (PI3K)/protein kinase B (Akt)/mTOR pathway and improves brain function after traumatic brain injury [6]. Also, NaHS can protect against septic cardiomyopathy by inhibiting autophagy through the AMPK/mTOR pathway [7]. Therefore, we speculated that NaHS may be involved in the regulation of mTOR signaling.

MicroRNAs (miRNAs) are a class of small endogenous noncoding single-stranded RNAs with a length of about 22 nt. miRNAs play an important regulatory role in the development of many cardiovascular diseases [8, 9]. Our previous experimental results revealed that miR-133a-3p was differentially expressed in LPS-treated cardiomyocytes. miR-133a-3p can promote the progression of thyroid cancer by regulating the PI3K/AKT/mTOR pathway [10]. Guanidinoacetic acid regulated myogenic differentiation and muscle growth through the miR-133a-3p and miR-1a-3p comediated Akt/mTOR/S6K signaling pathway [11]. Recently, some studies indicated autophagy participated in the molecular mechanism of body damage via measuring miRNAs, ATP, mTOR, AMPK, Beclin-1, LC3I/II, and P62. Cadmium decreased mTOR, increased miR-25-3p, LC3II, and Beclin-1, and caused liver damage and autophagy [12]. Energetic impairment as well as miR-9-5p and mTOR decreases occurred in cadmium exposure-caused myocardial injury and autophagy [13]. Energy metabolism disorder, mTOR, Beclin-1, LC3b, and P62 took part in the molecular mechanism of chlorpyrifos-induced liver damage and autophagy [14]. However, it is an unclear relationship between NaHS and miR-133a-3p in the development of myocardial injury in LPS-induced models. Therefore, this study used LPS-treated H9c2 cells to mimic sepsis-induced myocardial damage [3]. We aimed to elucidate the protective effect of NaHS on myocardial injury through miR-133a-3p-regulated autophagy and to provide new insights for basic research and clinical treatment of sepsis-induced myocardial injury.

## 2. Materials and Methods

### 2.1. Grouping and Model Building

H9c2 cells were purchased from Procell Life Science & Technology Co., Ltd (Wuhan, China, CL-0089). H9c2 cells were cultured to logarithmic growth phase and inoculated in 6-well plates with a concentration of 1 × 10^5^/mL. The cells were cultured overnight, and the old medium was removed when the cells reached a proportion of 70–80% in the wells. The medium was replaced according to different groups (each group contained three samples): (1) control group: cultured in a fresh medium for 24 hours; (2) LPS group: cultured in a medium containing 10 *μ*g/mL of LPS for 24 hours; (3) LPS + NaHS group: cultured in a medium containing 10 *μ*g/mL of LPS + 200 *μ*M NaHS for 24 hours; and (4) LPS + NaHS + miR-133a-3p inhibition group: in this group, siRNA interference was conducted to inhibit miR-133a-3p expression, and the cells were incubated for 36 hours in a fresh complete medium without double antibodies. Following siRNA transfection, the cells were then treated with 10 *μ*g/mL of LPS + 200 *μ*M NaHS for additional 24 hours. The concentrations of NaHS and LPS used were selected based on the data from literature and previous experiments [15–17].

### 2.2. Cell Transfection

Transfection was performed with riboFECT™ CP Transfection Reagent (RiboBio, China, R10035.7). Transfection Reagent was used according to the instructions. A density of 5 × 10^4^ to 1 × 10^5^ cells was inoculated in 12-well plates containing the appropriate amount of complete medium. Transfection Reagent T was added at a concentration of 100 nM to cells when the cell proportion reached 30–50% of the wells. The cells were incubated with fresh complete medium without double antibodies at the time of transfection. The plates were incubated at 37°C in an incubator with CO_2_ for 36 h. Afterwards, the transfected cells were treated with dosing for 24 h.

### 2.3. ATP Assay

ATP assay kit (Beyotime, China, S0026) was used to perform ATP assay on the cells according to the kit instructions.

### 2.4. RNA Extraction and Real-Time Quantitative PCR

RNA extraction and real-time quantitative PCR (RT-qPCR) procedures were performed according to the instructions of the kits. Total RNA was isolated from cells using RNAiso (Takara, Japan, D312). mRNA expression was assessed by RT-qPCR using a first-strand cDNA synthesis kit (Takara, Japan, RR036A) and a qPCR kit (Takara, Japan, RR390A). The expression levels of miR-133a-3p, mTOR, and AMPK were detected. For miR-133a-3p, U6 was regarded as the internal reference, and GAPDH was regarded as the internal reference for the other genes. The primer sequences for these genes are listed in Table 1. qPCR conditions were set as follows: 95°C for 2 min, 95°C for 5 s, 55°C for 30 s, and 72°C for 1 min, with a total of 40 cycles. Triplicate analysis was conducted for all samples. The relative expression levels of genes were calculated by using the 2^−△△Ct^ method.

### 2.5. Western Blot (WB)

The total protein was extracted, and the protein concentration was determined by using a protein concentration detection kit (Takara, Japan, T9300A). Protein samples were mixed with a buffer at a ratio of 4 : 1, and protein concentrations were kept constant at 4 *μ*g/*μ*L. Protein samples were separated using a 4–20% SDS-PAGE gradient prep gel (Kingsley, China), and the separated proteins were transferred onto PVDF membranes. The primary antibodies of mTOR, AMPK, LC3I/II, P62, and Beclin-1 were diluted with 3% bovine serum albumin at a ratio of 1 : 1000 to 1 : 2000. They were purchased from CST (USA). The membranes were added with primary antibodies and incubated at 4°C overnight. The next day, the secondary antibody (diluted to 1 : 5000 with the closure solution, Santa Cruz, USA) was added to the samples for incubating for 1 h. The enhanced chemiluminescence reagent exposure solution was mixed at a ratio of 1 : 1 of liquid A: liquid B and evenly covered on the membrane for 1 min. Finally, the membrane was examined by using the exposure meter.

### 2.6. Statistical Analysis

The data were analyzed using SPSS (version 20.0). All data were presented as mean ± standard deviation ($\bar{x}$ ± *s*). The means of multiple groups were compared by one-way ANOVA, and a LSD-t test was performed between groups. A p value <0.05 was considered as statistically significant.

## 3. Results

### 3.1. The Effect of NaHS on ATP and mir-133a-3p Levels in LPS-Treated Rat Cardiomyocytes

**Figure 1:**
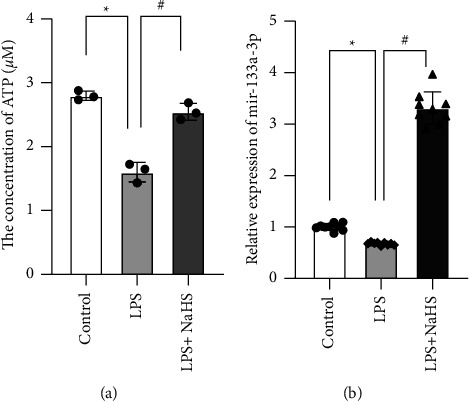
The expression levels of ATP and miR-133a-3p. (a) Total cellular ATP levels were determined by using an ATP assay kit. Relative ATP level in each group. (b) We measured the expression level of miR-133a-3p by qPCR. ^*∗*^*P* ≤ 0.05 compared with the control group and ^#^*P* ≤ 0.05 compared with the respective LPS-treated group.

ATP and miR-133a-3p levels were significantly lower in the LPS group than those in the control group (*P* < 0.001 and *P* = 0.001, respectively). However, ATP and miR-133a-3p levels were significantly increased in the LPS + NaHS group compared with those in the LPS group (*P* < 0.001 and *P* < 0.001, respectively) (Figure 1).

### 3.2. The Effect of NaHS on Autophagy-Related Factors in LPS-Treated Rat Myocardium

**Figure 2:**
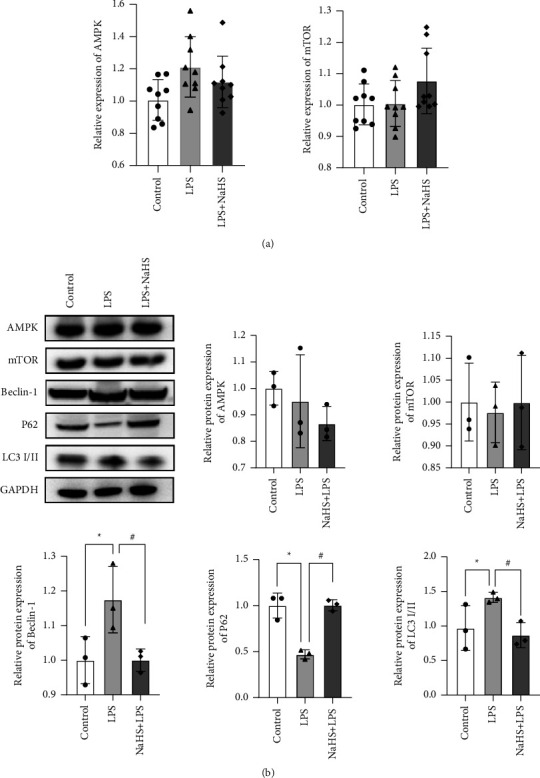
QPCR assay and WB were used to detect the expression of related indicators. (a) AMPK and mTOR expressions were determined by qPCR. (b) Western blotting with densitometric analysis of AMPK, mTOR, Beclin-1, P62, and LC3I/II. ^*∗*^*P* ≤ 0.05 compared with the control group and ^#^*P* ≤ 0.05 compared with the respective LPS-treated group.

The transcript levels and protein levels of AMPK and mTOR were altered after LPS and NaHS treatment, but the differences were not statistically significant between the groups (*P* > 0.05). For protein expression levels of Beclin-1, LC3I/II, and P62, we observed increased expression levels of Beclin-1 protein and LC3I/II protein, but decreased expression levels of P62 protein after LPS treatment, compared with the control group (*P* = 0.023, 0.048, and *P* < 0.001, respectively). However, in the LPS + NaHS group compared with the LPS group, Beclin-1 protein and LC3I/II protein expressions were significantly decreased and P62 protein expression was significantly increased (*P* = 0.023, 0.022, and *P* < 0.001, respectively) (Figure 2).

### 3.3. NaHS-Regulated ATP Expression via miR-133a-3p

**Figure 3:**
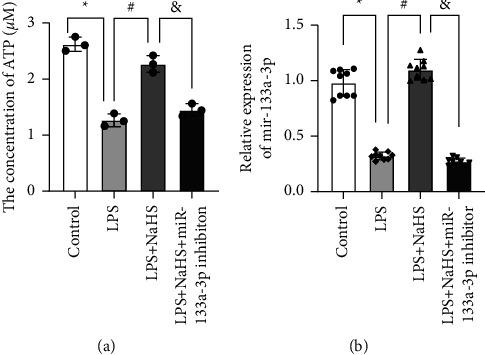
The expression levels of ATP and miR-133a-3p. (a) Total cellular ATP levels were determined using an ATP assay kit. Relative ATP levels in each group. (b) We measured the expression level of miR-133a-3p by qPCR. ^*∗*^*P* ≤ 0.05 compared with the control group, ^#^*P* ≤ 0.05 compared with the respective LPS-treated group, and ^&^*P* ≤ 0.05 compared with the LPS + NaHS group.

Compared with the control group, the levels of ATP and miR-133a-3p were decreased in the LPS group (*P* < 0.001 and *P* < 0.001, respectively). Compared with the LPS group, ATP and miR-133a-3p expressions were significantly increased in the LPS + NaHS group (*P* < 0.001 and *P* < 0.001, respectively). After treatment with NaHS and miR-133a-3p inhibition, ATP and miR-133a-3p expressions were significantly decreased in the LPS + NaHS + miR-133a-3p inhibition group (*P* < 0.001 and *P* < 0.001, respectively) (Figure 3).

### 3.4. NaHS-Regulated Autophagy via miR-133a-3p

**Figure 4:**
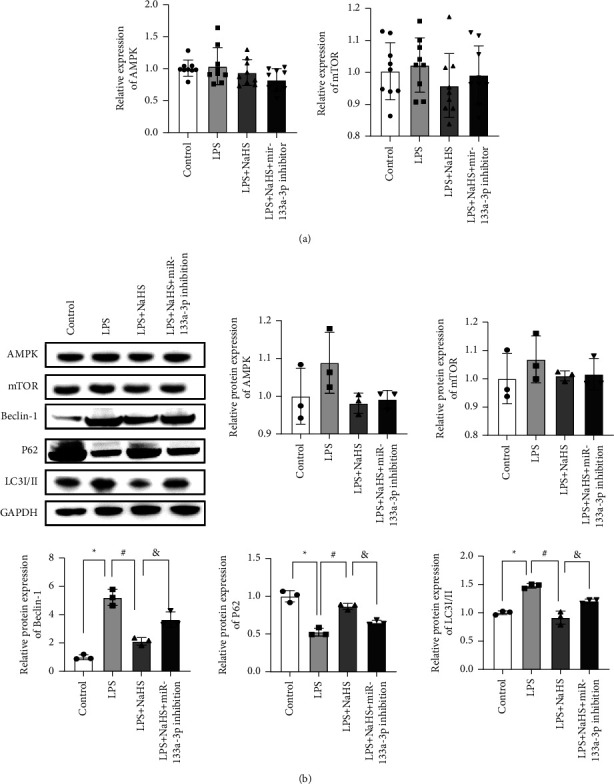
QPCR assay and WB were used to detect the expression of related indicators. (a) AMPK and mTOR expressions were determined by qPCR. (b) Western blotting with densitometric analysis of AMPK, mTOR, Beclin-1, P62, and LC3I/II. ^*∗*^*P* ≤ 0.05 compared with the control group, ^#^*P* ≤ 0.05 compared with the respective LPS-treated group, and ^&^*P* ≤ 0.05 compared with the LPS + NaHS group.

We found no significant differences in the transcriptional and protein levels of mTOR and AMPK among the control, LPS, LPS + NaHS, and LPS + NaHS + mir-133a-3p groups (*P* > 0.05 and *P* > 0.05, respectively), which suggested that AMPK and mTOR may be not involved in autophagy in myocardial damage induced by LPS. Compared with the control group, P62 protein expression was decreased, but Beclin-1 and LC3I/II protein expressions were increased in the LPS group (*P* < 0.001, *P* < 0.001, and *P* = 0.003, respectively). While P62 protein expression was increased, Beclin-1 and LC3I/II proteins were decreased after NaHS treatment compared with the LPS group (*P* < 0.001, *P* < 0.001, and *P* = 0.005). However, the expression of P62, Beclin-1, and LC3I/II protein was reversed (P62 expression was decreased and Beclin-1 and LC3I/II expressions were increased) by miR-133a-3p compared to that in the LPS + NaHS group (*P* = 0.001, *P* = 0.012, and *P* = 0.010, respectively) (Figure 4).

## 4. Discussion

ATP is the pivotal energy-carrying molecule within every cell, and the inability to sustain sufficient ATP levels can prove to be a critical factor in numerous diseases [18]. In intact HepG2 cells, LPS decreased mitochondrial membrane potential and cellular ATP content [19]. Similarly, in mouse bone marrow-derived macrophages activated with LPS, total ATP levels decreased significantly [20]. In this study, we found that the ATP level was significantly lower in the LPS group than that in the control group. H_2_S can provide electrons for mitochondria through the electron transport chain: quinone oxidoreductase (SQR) and mitochondrial complex II, which stimulates oxidative phosphorylation and increases mitochondrial ATP production [21]. NaHS acts as a H_2_S donor and can provide H_2_S to organisms for producing the corresponding biological functions. The present study confirmed that the ATP content of LPS and NaHS-cotreated rat cardiomyocytes was higher than that of rat cardiomyocytes treated with LPS alone.

NaHS can regulate apoptosis and autophagy in various ways. Previous studies have shown that NaHS can inhibit LPS-induced injury in A549 lung cancer cells by increasing cell viability, transmembrane electrical impedance, and expression of occluded small-band proteins and decreasing CRP levels [22]. NaHS can restore mitochondrial function and inhibit autophagy through the PI3K/Akt/mTOR signaling pathway to ameliorate traumatic brain injury [23]. In addition, NaHS can also activate autophagy to protect colon cells in ulcerative colitis [23] and protect against LPS-induced AKI by promoting autophagy [24]. However, what mechanisms of NaHS play an important role in LPS-induced myocardial damage remains unclear. Our study found that P62 protein expression was decreased and the expressions of Beclin-1 and LC3I/II proteins were elevated in rat cardiomyocytes after LPS treatment, whereas increased P62 protein expression and decreased Beclin-1 and LC3I/II protein expression were observed in LPS and NaHS-cotreated cardiomyocytes with NaHS treatment. The simultaneous increase in ATP levels was detected. Therefore, we hypothesized that NaHS may improve myocardial injury by reducing ATP consumption. These results provide direction for future experiments and further research into the correlation between ATP and autophagy.

The core lipid kinase complex of autophagy contains BECN1, phosphatidylinositol-3-kinase catalytic subunit type 3 (PIK3C3), and phosphoinositide 3-kinase regulatory subunit 4 (PIK3R4) [25], which plays a key role in autophagy activation. The process of autophagy is regulated by more than 30 regulatory genes and multiple pathways. For example, the PI3K-AKT-mTORC1 pathway-induced autophagy was observed in isolated mouse fibroblasts [26]. The kinase ULK1 can activate autophagy by phosphorylating Beclin-1 that binds to ligands [27]. Phosphatidylinositol-3-kinase complex can regulate autophagy through MAPK8/JNK1 activation. Bcl2 inhibits autophagy by interacting with BECN1. LC3 is an autophagy-associated core protein that is required for autophagosome formation and maturation [28]. LC3 is present in the cytoplasm as LC3I, and LC3II is formed through binding LC2I to phosphatidylethanolamine after autophagy, which specifically binds to phagosomes (precursors of autophagosomes) and induces autophagy [29]. Different autophagic pathways may differ under different cellular or survival conditions. Our experiments demonstrated that AMPK/mTOR pathway-related proteins were little changed in LPS-induced rat cardiomyocytes, while P62, LC3I/II, and Beclin-1 protein levels were significantly altered. Moreover, the expression of these proteins could be regulated by NaHS to ameliorate the myocardial damage. H_2_S attenuated sleep deprivation-induced cognitive impairment by reducing excessive autophagy of hippocampal SIRT 1 in WISTAR rats [30]. H_2_S protected retinal pigment epithelial cells from oxidative stress-induced apoptosis and affected autophagy [31]. In addition, H_2_S attenuated acute myocardial ischemic injury by modulating autophagy and inflammatory response under oxidative stress [32].

Noncoding RNAs (ncRNAs) play a wide range of regulatory roles in various diseases such as cardiovascular disease and are considered major players in physiological and pathological processes [33–35]. miR-133a-3p, a muscle-enriched miRNA, is highly expressed in the heart. Several studies have previously demonstrated that miR-133a-3p expression levels are significantly elevated in the circulation of patients with coronary artery disease, making it a promising blood biomarker for coronary artery disease [36, 37]. Chen et al. found that miR-133a expression was elevated in sepsis and could regulate sepsis-induced organ dysfunction and inflammatory responses by targeting SIRT1 [38]. However, our study found that miR-133a-3p expression was decreased in LPS-treated cardiomyocytes, while the expressions of autophagy-related proteins were altered where Beclin-1 and LC3I/II expressions were increased and P62 expression was decreased. After NaHS treatment in rat cardiomyocytes, miR-133a-3p expression was elevated. Moreover, autophagy-related proteins were also altered after NaHS treatment, with decreased Beclin-1 and LC3I/II expressions and increased P62 expression. It may be related to different cell models, and apoptosis-related studies also showed similar results that miR-133a-3p inhibited apoptosis by binding downstream to the 3′ UTR structural domain of CDIP1 to reduce transcriptional levels [32]. The simulant of miR-133a-3p promoted the phosphorylation of AKT in HUVEC or H9c2 cells [39]. In our experiments, we found that autophagy-associated proteins were altered in cardiomyocytes after treatment with NaHS but reversed after inhibition of miR-133a-3p expression, suggesting that NaHS regulated autophagy in LPS-stimulated cardiomyocytes through miR-133a-3p and thus protected the myocardium. Although LPS-stimulated cardiomyocytes showed decreased ATP content and energy supply, the AMPK level was not affected. In spite of no activation in the AMPK/mTOR-related autophagic pathway, other autophagy-related proteins could regulate autophagy levels in cardiomyocytes.

In summary, miR-133a-3p expression was decreased in LPS-treated H9c2 cells and miR-133a-3p expression level was significantly upregulated after NaHS treatment, while the inhibition of miR-133a-3p expression reversed the altered expression of autophagy proteins. The abovementioned results demonstrated that NaHS could upregulate the level of miR-133a-3p and alter the expression levels of autophagy proteins. Meanwhile, we found that the expression of mTOR and AMPK was not significantly changed. Therefore, it could be suggested that NaHS had a protective effect on myocardial injury by promoting miR-133a-3p expression, thus inhibiting autophagy and restoring cellular ATP levels in cardiomyocytes. The present study provided a new direction for the mechanism and treatment related to sepsis-induced myocardial injury, and further studies on their direct targets will be explored in future experiments.

## Figures and Tables

**Table 1:** Primer sequence.

Primer name	Primer sequence (5′–3′)
miR-133a-3p upstream	CAGTGCGTGTCGTGGAGT
miR-133a-3p downstream	CCAGCTGGTCGTATCCAGT
U6 upstream	CTCGCTTCGGCAGCACA
U6 downstream	AACGCTTCACGAATTTGCGT
mTOR upstream	GGTGGAAAGCCGTTGTTGC
mTOR downstream	CAGTGAGTTCTTGCTGCTCCTAC
AMPK upstream	CCCTTGAAGCGAGCAACTATC
AMPK downstream	GGTCTTGAGGGTCACCACTGTA
GAPDH upstream	GGCACAGTCAAGGCTGAGAATG
GAPDH downstream	ATGGTGGTGAAGACGCCAGTA

## Data Availability

The data used to support the findings of this study are available from the corresponding author upon request.

## Additional Points

*Highlights*. (1) miR-133a-3p expression increased in models of LPS-induced cardiomyocyte injury. (2) NaHS reduced LPS-induced myocardial impairment in a miR-133a-3p dependent manner.
